# Computed Tomography Assessment of Healthy Elbow Joint Congruity in Dogs Being Affected by Pronation and Supination Angulation: A Cadaveric Study

**DOI:** 10.3390/ani15070921

**Published:** 2025-03-23

**Authors:** Vivienne Chantal Katharina Possiel, Nikolaus Hubertus Huels, Adriano Wang-Leandro, Holger Andreas Volk, Oliver Harms

**Affiliations:** 1 Clinic for Small Animal Medicine, University of Veterinary Medicine Hannover, Foundation, 30559 Hannover, Germanyholger.volk@tiho-hannover.de (H.A.V.); dr.o.harms@googlemail.com (O.H.); 2 Specialist Center for Small Animal Medicine, 30885 Langenhagen, Germany

**Keywords:** medial coronoid process disease, radio-ulnar incongruity, elbow dysplasia, dog, elbow flexion

## Abstract

The aim of this study was to determine the influence of limb positioning on computed tomographic (CT) measurement of radio-ulnar incongruity (RUI) in canines. The sample population was comprised of twenty thoracic limbs harvested from ten adult canine cadaveric specimens of medium-size breeds without concurrent orthopedic disease. CT scans of each elbow were performed using two different flexion angles (90° and 135°) in combination with five different supination and pronation angles (−35°, −15°, 0°, 15° and 35°). The limbs were aligned using a custom-made fixation device. For each scan, RUI was measured at the apex (AMC) and base (BMC) of the ulnar medial coronoid process and compared with each elbow joint in the study. As the values followed a normal distribution and the one-factorial analysis of variance produced a significant result, a Dunnett post hoc test was performed to compare the individual test conditions pairwise for significant differences. The results show no significant differences in the measurement of RUI between 90° and 135° of elbow flexion. The RUI measured at the AMC showed significant maximum deviations (*p* < 0.001) of 18.14% at −15° and 15° and 31.31% at −35° and 35°. Measurement of RUI at the BMC showed no significant differences at 15° of pronation; 15° of supination resulted in small deviations of 5.51% (*p* < 0.001). Angulation of 35° resulted in average maximum deviations of 11.51% (*p* < 0.001). Supination resulted in greater changes in the RUI than pronation. This study concludes that pronation and supination affect the congruity of the radio-ulnar joint space, most evident at 35°. These findings should be considered during the positioning of the forelimbs for diagnostic purposes.

## Simple Summary

Elbow dysplasia (ED) is a highly clinically relevant disease, as it is a common cause of thoracic limb lameness in young medium-to-large-breed dogs. The incongruity between the radius and ulna is discussed as a decisive pathomechanism, which can be influenced by various factors. This study examines changes in pronation and supination angulation in computed tomography imaging in order to further our understanding for diagnostic purposes of ED.

## 1. Introduction

Elbow dysplasia (ED) is a hereditary developmental disorder in dogs, first defined by the International Elbow Working Group in 1993. It includes fragmentation of the medial coronoid process (FMCP), Osteochondritis Dissecans (OCD), Sub-Trochlear Sclerosis (STS), Ununited Anconeal Process (UAP), Medial Compartment Disease (MCD) and Elbow Incongruency (EI) [1,2,3,4]. ED is one of the most common causes of forelimb lameness in young medium-to-large-breed dogs [3] and occurs bilaterally in up to 35% of cases [5,6,7]. All forms of ED lead to degenerative changes and osteoarthritis [8,9]. FMCP occurs much more frequently than OCD of the medial condyle of the humerus or UAP and only slightly more frequently than EI [10].

Two types of incongruity have been defined: humero-ulnar and radio-ulnar [11]. In cases of humero-ulnar incongruity, the trochlear notch of the ulna has an abnormal shape, which prevents optimal articulation with the humeral condyle. Radio-ulnar incongruity (RUI) is defined as a step formation between the articular surfaces of the radius and ulna [1,11,12].

RUI has been reported to occur concomitantly with medial coronoid process disease (MCPD) in up to 60% of clinical cases and has been shown to be associated with the severity of medial compartment pathology [13]. Static positive RUI (short radius) results in a load shift from the radial head to the medial coronoid process [2,14], leading to mechanical overloading of the medial articular compartment, cartilage damage, microfractures and ultimately FMCP [3,15,16,17].

Dynamic RUI has been proposed as an explanation for cases of MCPD in which RUI was absent when dogs were positioned in dorsal recumbency and were under sedation [2,4,18,19,20,21,22]. The limitation between physiological and pathological incongruity has not yet been conclusively investigated. A major challenge is to decipher this dynamic incongruity. The elliptical shape of the ulnar trochlear notch present in healthy dogs is consistent with a bicentric distribution of weight forces [23]. During the various phases of a dog‘s gait cycle, the joint does not always seem to be completely congruent. In fact, there are physiological variations, and some parts of the joint appear to be more congruent during weight bearing than others during flexion [24].

Previous studies have recognized that the RUI is significantly affected by changes in limb position in terms of flexion and extension angulation and maximum physiologically pronation and supination angulation [25,26,27,28,29,30,31]. However, the results of previous studies must be verified by CT elbow congruity assessment using standardized pronation and supination angulations. The aim of our study is to investigate the effects of standardized positional changes in the assessment of elbow joint congruity.

Because the minimal axial rotation of the ulna was expected during pronation and supination of the antebrachium, our hypothesis was that RUI would not be affected by supination and pronation of 15° and significant differences would be found at 35°. We also hypothesized that changes in joint flexion would have a significant influence on the measurement of RUI.

## 2. Materials and Methods

### 2.1. Inclusion Criteria

Ten dogs were included in the study, representing a total of twenty thoracic limbs. The dogs belonged to medium-to-large breeds, including one intact male, four neutered males, two intact females and three neutered females. Mean age was 8.0 ± 4.13 years and mean weight was 26.73 ± 7.94 kg. The breeds included in the study were Labrador Retriever, Golden Retriever, Rhodesian Ridgeback, Shar Pei, White Swiss Shepherd, German Shorthaired Pointer, Greyhound and mixed breed.

All of these dogs were euthanized at the University of Veterinary Medicine Hannover, Germany, for reasons unrelated to the study, and the written consent for image acquisition and use of data for research purposes was given by the owners. None of the dogs had clinical orthopedic disease. Dogs with previously diagnosed elbow joint disease or arthritis of the elbow joint were excluded from the study.

The thoracic limbs were amputated at the shoulder joint immediately after euthanasia, stored at 4 °C and examined within 48 h.

### 2.2. Positioning Aids

**Figure 1:**
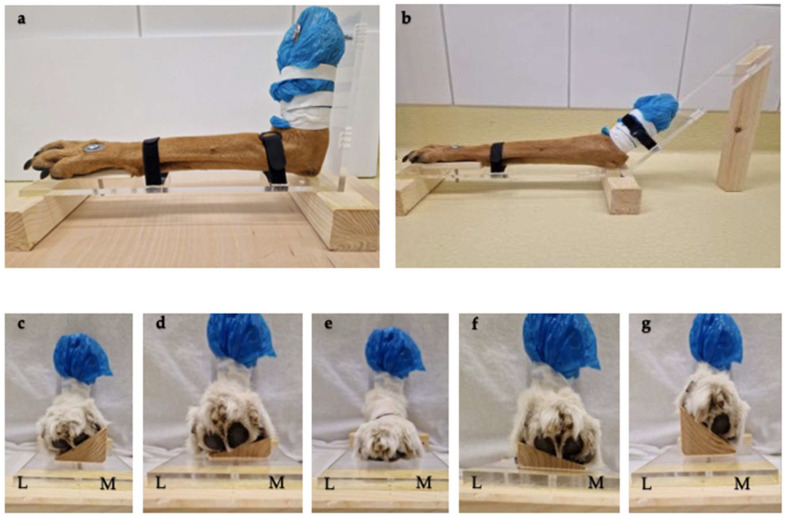
(**a**) Limb in 90° positioning aid. (**b**) Limb in 135° positioning aid. (**c**–**g**) Limb in −35° + −15° supination, 0°, 15° + 35° pronation (view from left to right). L = lateral, M = medial.

Positioning aids were made of radiolucent materials, polymethyl methacrylate and wood, to avoid artifacts. With the aid of this device, the limb could be accurately angled at both 90° and 135° (Figure 1a,b).

During the study, the thoracic limbs were sequentially immobilized in both positioning aids. A 10 cm screw was inserted through the 2nd and 3rd metacarpals, and a 4 cm diameter washer was used to provide the necessary pressure. In addition, the limb was fixed to the back wall of the positioning aid with another 10 cm screw through the proximal diaphysis of the humerus. Velcro straps (material: nylon; 2 cm × 15 cm) provided additional fixation.

To image the limb at different supination and pronation angles, the limb was repositioned after each individual CT scan. This was accomplished with wooden wedges angled at 15° and 35°. These wooden wedges were screwed under the limb at the level of the metacarpals in conjunction with the positioning aid (Figure 1c–g). The washers allowed controlled pressure to be applied to achieve the desired supination and pronation of the elbow.

### 2.3. Image Acquisition

CT scans of the elbows were performed using a spectral detector CT (Philips IQon Spectral CT, Philips Healthcare Germany). For each elbow, raw data were acquired using a helical acquisition with a slice thickness of 0.67 mm, a pitch of 0.399 and a rotation time of 0.75 s for each different position. The KVp was set at 120, with an effective mAs of 80 using a bone reconstruction filter and a field of view of 100 mm. The images were displayed on a window width and level of 2000 HU and 800 HU, respectively. Each elbow was then subjected to the experimental conditions described below.

The sequences were performed with each limb in a neutral position at 0°, 15° and 35° pronation and 15° and 35° supination. These 5 scans were performed using the two different flexion angles of 90° and 135°.

### 2.4. RUI Measurements

**Figure 2:**
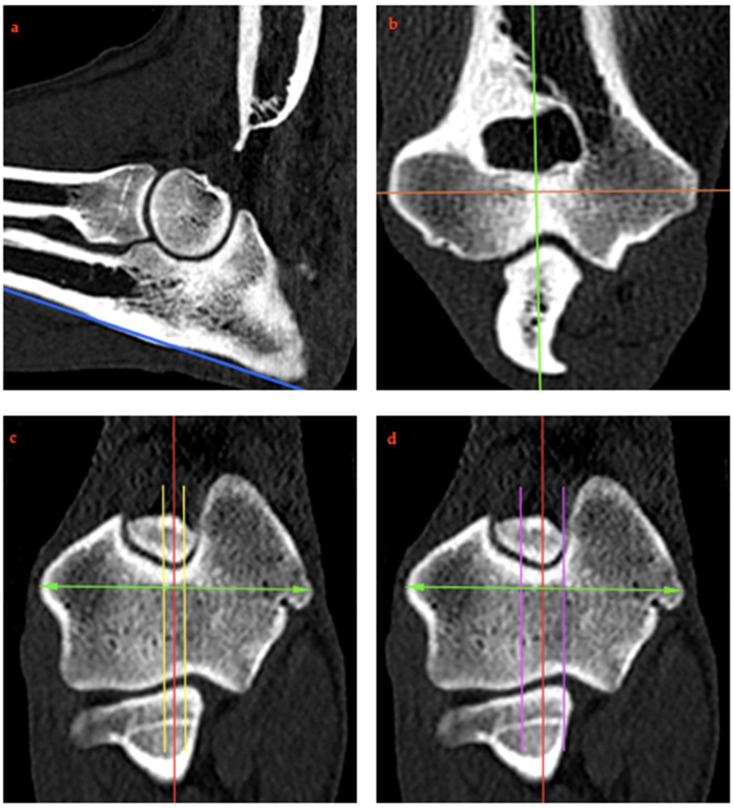
(**a**–**d**) Image reconstruction: A midline sagittal image of the elbow was obtained centrally by first aligning the dorsal plane to the caudal cortex of the olecranon ((**a**) blue line), the sagittal plane to the longitudinal axis through the supratrochlear foramen of the humerus ((**b**) green line), and aligning the transverse plane to bisect the humeral condyle at its widest point (orange line). The central sagittal image was then obtained in the transverse plane (red line). Two lateral and two medial compartment parasagittal images were obtained by measuring the transcondylar distance in the transverse plane (green arrows) with an offset of 5% ((**c**) yellow line) and 10% ((**d**) purple line) of the humeral condyle relative to the midline sagittal image (red line).

Dicom images were evaluated using Horos™ (version 3.3, The Horos Project; Purvis, Annapolis, MD, USA) as 3D multiplanar images showing sagittal, transverse and dorsal planes simultaneously. To obtain comparable measurements, the method of Burton and colleagues for standardizing reconstructions was followed [27]: A midline sagittal image of the elbow was generated centrally by aligning the dorsal plane with the caudal cortex of the olecranon (Figure 2a), the sagittal plane with the long axis through the supratrochlear foramen of the humerus (Figure 2b), and the transverse plane so that the humeral condyle was bisected at its widest point (Figure 2c).

A sagittal slice was taken through the supratrochlear foramen of the humerus at the center of its long axis, maintaining the orientation described above. The humerus was chosen as a landmark because its position was the only bone that did not change in the different positions. This method provided a consistent slice orientation for the measurement of each elbow in the positions described previously.

In addition to obtaining the central sagittal image, two lateral and two medial parasagittal images were obtained. These corresponded to a lateral and medial offset of 5% and 10%, respectively, of the total humeral condyle width with respect to the central sagittal image as defined in the transverse view of the humeral condyle (Figure 2c,d). These parasagittal images were created solely for the RUI BMC to investigate the effect of a deviation in the shift of the most central orientation to the lateral or medial direction on the RUI at this area.

**Figure 3:**
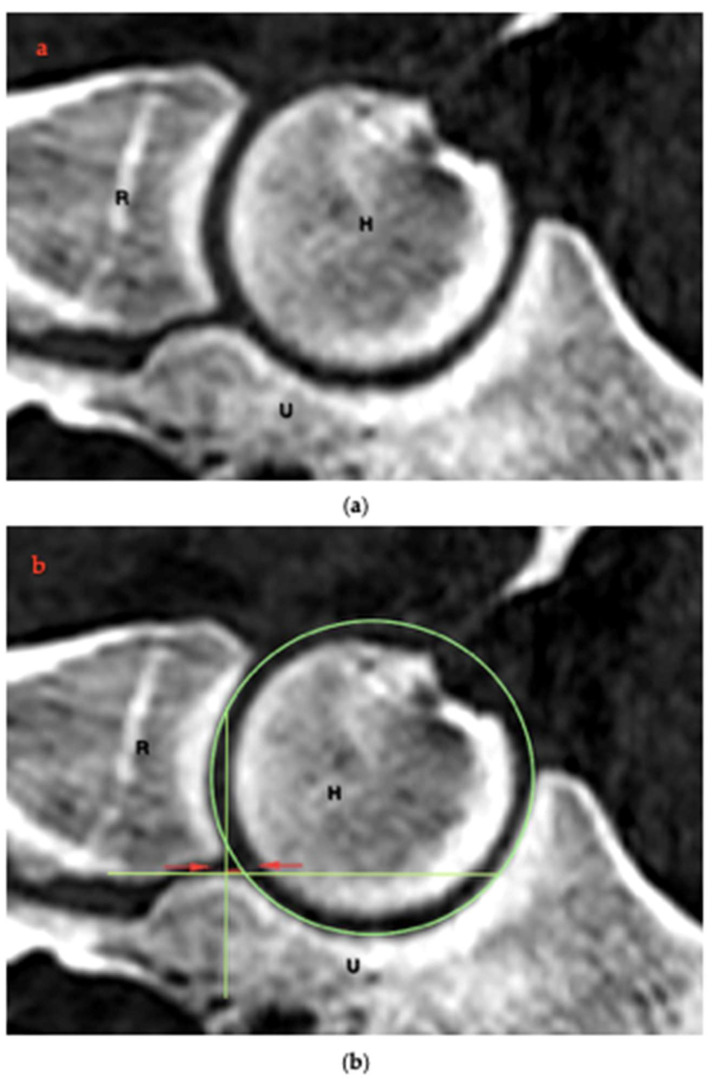
(**a**,**b**) Sagittal CT image of the elbow (**a**) showing the RUI BMC ((**b**), the red line between red arrows) using a circular graphical construct (green circle and green lines marking the bone surface of the radius and ulna). H: humerus, R: radius, U: ulna.

The measurement of the RUI was modified from the method described by Kramer and colleagues [4]. For this purpose, the previously reformatted central sagittal plane was used to measure the RUI at the BMC (Figure 3a). RUI was defined as the distance between the subchondral bone surfaces of the radio-ulnar articulation. These bone surfaces were marked with single-pixel-thickness lines at their proximal edge using the same computer analysis program; on the sagittal plane image, a circular graphic construct was used to further delineate the ventral edge of the ulnar notch, which aided in the accurate placement of the ulnar coronoid base line. In addition, another line was marked at right angles along the radial incisure of the ulna. The distance between these lines was defined as RUI BMC (Figure 3b; red line). The RUI BMC was determined for each individual scan (neutral position 0°, pronation 15° and 35° and supination 15° and 35°), both in the central sagittal section and in the medial and lateral parasagittal sections (Figure 2c,d).

**Figure 4:**
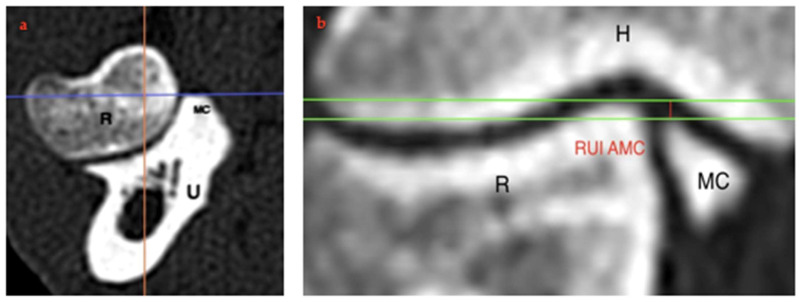
(**a**) Transverse plane of the radio-ulnar joint for localization of the AMC (blue line) using the centre line (orange line) to be assessed. (**b**) Reformatted CT images in the dorsal plane. Exemplary representation of the change in RUI AMC (red line) in the neutral position (0°). H: humerus, R: radius, U: ulna, MC: medial coronoid.

To measure the RUI at the AMC, the transverse plane was positioned at the most distal point of the medial coronoid (Figure 4a; blue line). On the dorsal plane image, two single-pixel-thickness lines were used to mark the subchondral bone surfaces at their proximal edge. The distance between these lines was defined as the RUI AMC (Figure 4b; red line). The RUI AMC was also measured in the neutral position and for each pronation and supination angulation. All measurements were performed twice at 3-week intervals by a single operator, and the mean values were used for statistical analysis.

### 2.5. Statistical Analysis

For statistical data analysis, the software RStudio™ (version 3.3.0; Posit PBC, Boston, MA, USA) was applied. The data were graphically tested for normal distribution using histograms. Analysis of variance was chosen as the appropriate statistical method to compare the different measurements under each test condition for each region; *p*-values < 0.05 were considered statistically significant. As the values followed a normal distribution and the one-factorial analysis of variance produced a significant result, a Dunnett post hoc test was performed to compare the individual test conditions pairwise for significant differences.

Because of the various confounding factors including differences in size and weight, absolute values were converted to percentage change rates for each measurement.

## 3. Results

**Figure 5:**
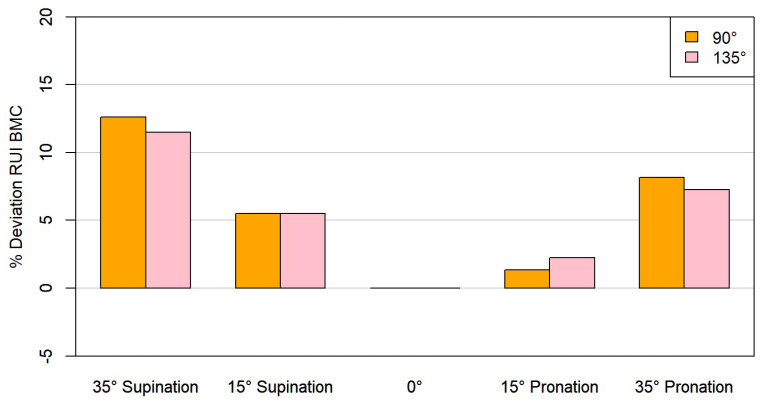
Representation of the RUI BMC at standardized supination and pronation angles compared to 90° and 135° elbow angles.

**Figure 6:**
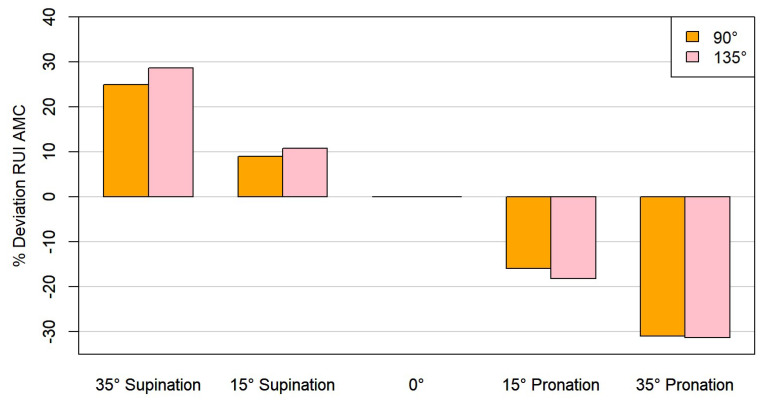
Representation of the RUI AMC at standardized supination and pronation angles compared to 90° and 135° elbow angles.

RUI was not influenced by different flexion angles (90° and 135°) (Figure 5 and Figure 6).

No significant differences were found when evaluating the RUI BMC at 15° pronation relative to the neutral position (135°: *p* = 0.065, 90°: *p* = 0.492). All other position changes resulted in statistically significant deviations. At 135° elbow flexion with 35° pronation, the mean percentage deviation (mean %) increased by 7.27%; 15° supination resulted in average deviations of 5.51% and 35° supination of 11.51% (Table 1). Overall, the changes were slightly more pronounced in supination than in pronation (Figure 5).

No significant differences were found when evaluating the RUI AMC at 15° supination relative to the neutral position at 90° (*p* = 0.080). All other position changes resulted in statistically significant deviations for the RUI AMC (*p* < 0.05) (Table 1).

The RUI AMC decreased with pronation and increased with supination. There was an average increase of 10.75% at 15° supination and a 28.67% increase at 35° supination. The average decrease in the RUI was 18.14% at 15° pronation and 31.31% at 35° pronation (Figure 6).

The RUI BMC measured in the neutral position in parasagittal slices induced statistically significant deviations at each lateral and medial displacement (*p* < 0.001).

The RUI BMC increased in mean percentage deviation (mean %) by 3.68% at 5% lateral displacement and 8.57% at 10% lateral displacement. At 5% medial displacement, the RUI decreased by an average of 4.78%, and at 10% medial displacement, the RUI decreased by 9.67% (Table 2).

## 4. Discussion

CT imaging of the elbow is an important step in diagnosing canine ED. CT has proven its diagnostic superiority over X-ray examination in detecting MCD in dogs [10]. The influence of limb positioning on CT measurement of RUI in canines might influence ED diagnosis. The current cadaveric study showed that pronation and supination affect the congruity of the radio-ulnar joint space, most evident at 35°, but no significant differences in joint space dimensions between 90° and 135° elbow flexion were seen. These findings should be considered during positioning of the thoracic limbs for diagnostic purposes.

Measurements of the RUI AMC showed clear deviations between the neutral position measurement and the measurements at different supination and pronation angles. Slightly larger RUI differences were measured in pronation than in supination. These differences reached up to 31.31% and can be explained by the raising and lowering of the apex of the medial coronoid in pronation and supination [29]. Based on the results obtained, the measurement of the RUI AMC may impair the clinical assessment of joint congruity, as even small displacements of 15° lead to deviations of 18.14% on average in pronation.

The RUI BMC measurements showed significantly different results: At a low pronation of 15°, the deviations were so small that they were not statistically significant. More pronounced rotation of the limb resulted in statistically significant deviations. A challenge of this study was to obtain cross-sectional images that were at the exact same location, independent of changes in limb position. This limited how well RUI measurements could be compared. Saying this, the slice thickness was 0.67 mm limiting this bias.

The present study helps, by means of standardized pronation and supination angles, to characterize the degree of incongruity of the elbow joint that may be attributed to positional changes instead of pathological incongruity. The applicability of static RUI measurements for the evaluation of pathologic elbow incongruity remains questionable, as several studies have shown that dynamic incongruity occurs during motion and compressive loading [20,21,22]. For instance, an axial displacement of the radius and ulna of up to 1.0 mm in orthopedically healthy dogs [20] as well as a dynamic translocation of the radius of 0.7 mm in healthy dogs and 0.5 mm in dogs with dysplastic elbows have been reported [22]. Recently, Rohwedder and colleagues showed that the static RUI in healthy elbows did not correspond to the in vivo condition during loading and that a dynamic RUI of up to 0.6 mm occurred [21].

The current CT studies did focus on joint morphology depending on the different limb positions but lacked loading the limbs to simulate ground reaction forces generated during movement, which is influenced by body weight. This was not possible in the current set-up. RUI measurements are most likely a dynamic process, which varies depending on limb positioning and load during different gait paces [21,22,24]. A future study combining both compressive loading and positional shifts would be desirable.

Our study highlights the importance of measuring an accurately centered cross-sectional image. The measurement of parasagittal images with shifts of 5% and 10% showed slightly higher deviations medially compared to laterally. This effect on the measurement of the RUI is still limited, with a maximum of 9.67%. However, these measurements were made with the limb in a neutral position. If both pronation or supination of the limb is present and, in addition, measurements are not taken in the center but in the parasagittal region, these two errors may add up and lead to significant changes in RUI.

A limiting factor is the small number of samples and the lack of age, breed and size matching. The wide range of ages, breeds and body weights was deliberately chosen and considered an advantage to minimize possible errors due to breed- or age-related factors. To mitigate these effects, the absolute values for each elbow were converted to percentage rates of change of statistical analysis.

In the dogs studied, orthopedic diseases of the elbow were ruled out based on the pre-reported history of disease and negative findings on postmortem CT. No further diagnostic procedures to evaluate the synovial capsule and articular cartilage such as arthroscopy were performed. As CT only shows the subchondral bone and not the morphology, especially the articular cartilage thickness, this may affect the accuracy of the measurement [2,31,32,33]. Thus, the extent of RUI may not only be due to differences in bone architecture but also to differences in cartilage thickness. Other techniques such as MRI or contrast CT arthrography may be needed to more accurately measure RUI at the cartilage surfaces [34,35,36].

It would also be conceivable in the future to use analysis methods with artificial intelligence (AI) algorithms similar to a study from 2022, with a radiomics-based workflow applied to positron emission tomography (PET) imaging of mouse models that exploits image segmentation and the use of atlas first within a semi-automated process to assess the biodistribution of a new radiopharmaceutical and for the highly reproducible coregistration of images [37]. AI could positively influence research into the biomechanics of the elbow joint.

Another limiting factor is the degree of subjectivity in the measurement and interpretation of the CT images. This was minimized by having all CT measurements performed by a single operator. The resolution of the CT was deemed sufficient after numerous test runs, as small resolution-related errors are expected to cause only fractions of a millimeter difference in the measurements. As this study was performed exclusively on cadavers, factors such as radiation protection were not relevant with regard to the scan parameters.

Based on the knowledge gained regarding the visualization of RUI on CT, other diagnostic options such as arthroscopy should be considered for RUI measurement. Experimental radial shortening and lengthening studies of up to 3 mm showed a sensitivity of 0.97 and a specificity of 0.82 [31] and a sensitivity of 0.98 and a specificity of 0.89 [38] of arthroscopic estimation of RUI, respectively. The advantage of arthroscopy is the ability to visualize and palpate the RUI in the open joint. However, it should be noted that manipulation of the joint by abduction of the limb to widen the medial joint space and insertion of instruments [39] causes a conformational change in the joint [31,40], which may also influence visualization of the RUI in a similar way as pronation and supination on CT.

Extrapolating our results obtained in healthy elbows to dysplastic elbows is to a certain extent speculative. Similarly, the influence of muscle contractions and compression and bending moments generated by muscles and tendons during the gait cycle cannot be considered in such ex vivo studies. Therefore, further studies are needed to objectively compare the measurement of RUI in normal and dysplastic joints, including weight bearing during the gait cycle.

## 5. Conclusions

This study concluded that pronation and supination affected the congruity of the radio-ulnar joint space, most evident at 35°. Measuring the RUI AMC is not recommended due to the larger deviations in relation to the measurements of the RUI BMC.

RUI measurements were not influenced by different flexion angles (90° and 135°).

These findings suggest a possible effect on clinical diagnostic processes and should be considered during positioning of the thoracic limbs for diagnostic purposes. When positioning the canines, pronation and supination of the thoracic limbs should be avoided. Standardized elbow flexion is not necessary.

In terms of the clinical relevance of this study, it is imperative to consider the multifactorial nature of ED when interpreting the results. EI resulting from incorrect positioning of the thoracic limb does not necessarily lead to an erroneous diagnosis, as other factors, such as STS, OCD, UAP and FCP, must also be considered in the clinical diagnosis.

## Figures and Tables

**Table 1:** Results of the Dunnett pairwise post hoc test with the percentage deviation of the RUI BMC and AMC measured at 90° and 135° elbow flexion as a function of different supination and pronation angles.

RUI	Flexion	Compared Groups	Mean (%)	*p*-Value
BMC	90°	0° and 15° Pronation	1.36	0.492
		0° and 35° Pronation	8.18	<0.001
		0° and 15° Supination	5.51	<0.001
		0° and 35° Supination	12.62	<0.001
	135°	0° and 15° Pronation	2.25	0.065
		0° and 35° Pronation	7.27	<0.001
		0° and 15° Supination	5.51	<0.001
		0° and 35° Supination	11.51	<0.001
AMC	90°	0° and 15° Pronation	16.00	<0.001
		0° and 35° Pronation	30.97	<0.001
		0° and 15° Supination	9.02	0.080
		0° and 35° Supination	24.90	<0.001
	135°	0° and 15° Pronation	18.14	<0.001
		0° and 35° Pronation	31.31	<0.001
		0° and 15° Supination	10.75	0.027
		0° and 35° Supination	28.67	<0.001

**Table 2:** Listing of the mean percentage deviation of the RUI BMC in 135° and 90° elbow flexion compared between measured central sagittal images (0°) and measured lateral and medial parasagittal images.

Flexion	Compared Groups	Mean (%)	*p*-Value
135°	0% and 5% Medial	4.78	<0.001
	0% and 10% Medial	9.67	<0.001
	0% and 5% Lateral	3.68	<0.001
	0% and 10% Lateral	8.57	<0.001
90°	0% and 5% Medial	4.77	<0.001
	0% and 10% Medial	9.35	<0.001
	0% and 5% Lateral	4.05	<0.001
	0% and 10% Lateral	8.82	<0.001

## Data Availability

The datasets presented in this article are not readily available due to technical limitations. Request to access the datasets should be directed to vivienne.possiel@tiho-hannover.de.
